# Resistin promotes the abnormal Type I collagen phenotype of subchondral bone in obese patients with end stage hip osteoarthritis

**DOI:** 10.1038/s41598-017-04119-4

**Published:** 2017-06-22

**Authors:** Ashleigh M. Philp, Rebecca L. Collier, Liam M. Grover, Edward T. Davis, Simon W. Jones

**Affiliations:** 1 MRC-ARUK Centre for Musculoskeletal Ageing Research, Medical School, Queen Elizabeth Hospital, University of Birmingham, Birmingham, B15 2WB UK; 2 School of Chemical Engineering, University of Birmingham, Birmingham, B15 2TT UK; 3 The Royal Orthopaedic Hospital NHS Foundation Trust, Bristol Road South, Northfield, Birmingham B31 2AP UK

## Abstract

The purpose of this study was to determine the effect of adiposity on the architecture and composition of hip OA subchondral bone, and to examine the pathological role of adipokines. Femoral heads were collected from normal-weight or over-weight/obese patients with hip OA. Structural parameters of subchondral bone were determined by MicroCT and type I collagen *α*1/*α*2 ratio was determined by SDS PAGE and by qRT-PCR in *ex*-*vivo* bone explants. The serum concentration of adipokines was determined by Luminex. The effect of resistin on primary OA osteoblasts was determined by analysis of Wnt pathway signal transduction, bone nodule formation, and osteoblast metabolic activity. Subchondral bone from over-weight/obese hip OA patients exhibited reduced trabecular thickness, increased bone surface/bone volume ratio, and an increase in the Type I collagen *α*1/*α*2, compared to normal-weight hip OA patients. The serum concentration of resistin was higher in overweight/obese OA patients, compared to normal-weight OA patients. Stimulation of normal-weight bone explant with recombinant resistin increased the Type I collagen *α*1/*α*2 ratio. Stimulation of primary OA osteoblasts with recombinant resistin increased Wnt signalling activation, osteoblast metabolic activity, and bone nodule formation. Increased adiposity in hip OA patients is associated with altered subchondral bone architecture and type I collagen composition.

## Introduction

Osteoarthritis (OA) is the most common degenerative joint disease in the world and is typically characterised by the loss of articular cartilage and narrowing of the joint space^1, 2^. Historically, OA has been considered to be a disease solely of cartilage degeneration, which has resulted in cartilage being the prime focus of drug discovery research^3^. However, OA is now considered to be a disorder affecting all the tissues within the joint, including the subchondral bone. Indeed, evidence has emerged that pathologies identified in OA subchondral bone precede, and may contribute to, cartilage degeneration^4–6^.

For many years, increased adiposity has been recognised as a significant risk factor of OA. A study analysing the National Health and Nutrition Examination Survey (NHANES I) concluded that obese females were nearly four times more likely to experience OA symptoms than non-obese females, and male obese individuals were nearly five times more likely to develop OA symptoms than their normal- weight counterparts^7^. The increased prevalence of OA in both weight-bearing and non-weight bearing joints of obese individuals has led to the emergence of adipose-secreted cytokines^8^, termed adipokines, as candidate drivers of OA pathology. Adipokines have been purported to be key mediators of metabolic health^9–11^, being associated with metabolic syndrome related disorders^12^, and therefore provide a potential metabolic link between obesity and OA pathology.

To date, research into understanding the role of adipokines in OA joint pathology has predominantly focussed on the cartilage tissue by determining the effect of adipokines on chondrocyte phenotype. For example, using isolated primary human chondrocytes, Hui *et al*.^13^ discovered that leptin alone, and in synergy with IL-1*β*, induced the expression of catabolic factors metalloproteinases (MMP)-1 and MMP-13 with activation of p38, Erk, PI3K and Akt pathways. Leptin has also been demonstrated to increase the production of inflammatory mediators including IL-1*β*, IL-6, IL-8 and prostaglandin E2^14^. However, cartilage tissue is avascular and thus is unlikely to be directly affected by systemic increases in pathological levels of adipokines.

In contrast, subchondral bone is a highly vascularised tissue, and thus would be expected to be influenced by differential concentrations of circulatory adipokines reported in over-weight and obese individuals^9, 15, 16^. Despite this, comparative studies of subchondral bone pathology between OA patients of different adiposity has not been reported. Furthermore, very little is currently understood regarding the functional role of adipokines in mediating human OA bone pathology or their effect on the phenotype of human OA osteoblasts. We previously reported that there is a temporal relationship between serum levels of adipokines and biomarkers of bone remodelling in females with knee OA^17^, and more recent studies have found that the adipokines visfatin, resistin, and leptin modulate osteoblast proliferation and differentiation^18–20^, supporting the concept that adipokines and increased adiposity may impact on OA bone pathology.

Importantly, OA subchondral bone has been referred to as “sclerotic”, with reports of irregular trabecular architecture^21^, abnormal Type I collagen composition^22^, and dysregulated mineralisation^23^. Therefore, the aim of this study was two-fold. Firstly, to determine whether the trabecular structure and Type I collagen composition of subchondral bone differs between normal-weight hip OA patients and overweight/obese hip OA patients. Secondly to examine the effect of candidate pathological adipokines on the Type I Collagen composition of human hip OA bone and on the phenotype of primary human hip OA osteoblasts.

## Results

### Greater adiposity in patients with hip OA is associated with an abnormal Type I Collagen composition and altered structural parameters in the subchondral bone

**Figure 1:**
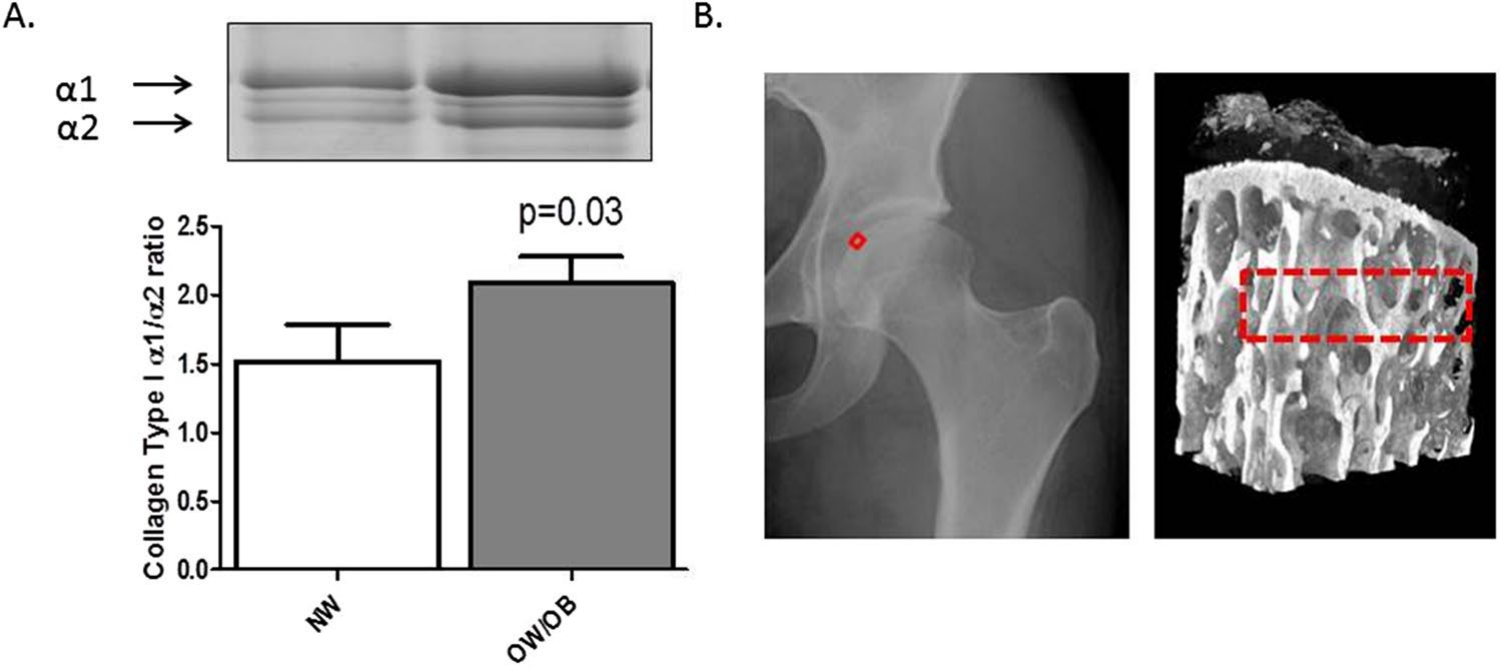
Comparison of the structural composition of femoral head subchondral bone in patients of different BMI cohorts. (**A**) The quantification of the *α*1/*α*2 Type I collagen ratio in femoral head subchondral bone. NW = normal-weight (n = 7), OW/OB = overweight/obese (n = 13). Bars represent mean *α*1/*α*2 values ± SEM as determined through ImageJ. The gel image is a representative coomassie-stained SDS PAGE gel of a NW OA and OW/OB OA femoral head, demonstrating the separation of *α*1/*α*2 type I collagen isoforms. (**B**) Representation of the area from the trabecular bone for micro-CT analysis. The area analysed was a 1 cm^3^ from the most medial aspect of the femoral head (as indicated by the red square on the femoral head). Dashed line box = region of interest which was reconstructed for bone parameter analysis.

Sclerotic subchondral bone in patients with knee OA has previously been attributed to an increase in the production of Type I collagen homotrimer, due to an increase in the relative proportion of *α*1 to *α*2 Type I collagen isoforms^22^. Therefore, we first used SDS PAGE to determine whether the relative proportion of *α*1 and *α*2 isoforms differed between subchondral bone samples from hip OA patients who were normal-weight (NW) compared to those who were over-weight/obese (OW/OB). Of significance we found that OW/OB subchondral bone samples exhibited a greater ratio of *α*1 to *α*2 (P < 0.05), compared to NW subchondral bone samples (Fig. 1A).

Next, we performed micro-CT analysis to determine the gross structural parameters of subchondral bone samples from the most medial aspect of the femoral head, as depicted in Fig. 1B. Comparing bone from OW/OB patients with NW patients, we found no significant difference in either the number (Tb.N) or the spacing (Tb.Sp) of trabecular. Furthermore, there was no difference in either the structural model index (SMI), degree of anistrophy (DA) or ellipsoid factor (EF), suggesting there were no rod or plate-like geometrical differences in the trabecular structure between subchondral bone samples from hip OA patients of varying BMI (Table 1). However, OW/OB bone exhibited significantly lower Trabecular thickness (TbTh.) compared with NW bone (0.29 ± −0.02 vs 0.34 ± 0.03 mm), and a significantly higher bone surface/bone volume ratio (7.53 ± 1.14 vs 5.9 ± 0.5 1/mm) (Table 1).

**Table 1:** Micro-CT analysis of OA femoral head subchondral bone samples from Normal-weight (NW, n = 6) and over-weight/obese (OW/OB, n = 6) patient cohorts.

Parameter	NW	OW/OB	p value
Gender (M:F)	3:3	3:3	—
Age (yr)	59 ± 10	57 ± 9	0.749
BMI	23.5 ± 1.1	33.5 ± 2.5	**0.007**
W:H ratio	0.87 ± 0.07	0.91 ± 0.08	0.303
Body Fat %	23.9 ± 7.3	38.1 ± 7.5	**0.008**
BV/TV	0.42 ± 0.04	0.43 ± 0.12	0.85
Tb.Th	0.34 ± 0.03	0.27 ± 0.04	**0.01**
Tb.N	1.25 ± 0.12	1.66 ± 0.62	0.14
Tb.Sp	0.47 ± 0.08	0.41 ± 0.25	0.61
BS/BV	5.91 ± 0.54	7.53 ± 1.14	**0.01**
SMI	−0.27 ± 0.5	−0.4 ± 0.95	0.76
DA	1.55 ± 0.1	1.45 ± 0.2	0.31
EF	−0.40 ± 0.14	−0.43 ± 0.22	0.16

Patient samples were excluded if a bone cyst was present within the 1 cm^3^ section of femoral head scanned. BV/TV = Bone volume/Total volume; TB.Th = Trabecular thickness; TB.N = trabecular number; TB.Sp. = trabecular separation; BS/BV = Bone surface/Bone volume; SMI = Structure Model Index; DA = Degree of Anistrophy, EF = Ellipsoid factor. Data expressed as mean ± SD.

### The Type I collagen composition of normal-weight hip OA subchondral bone is pathologically altered by the adipokine resistin

**Figure 2:**
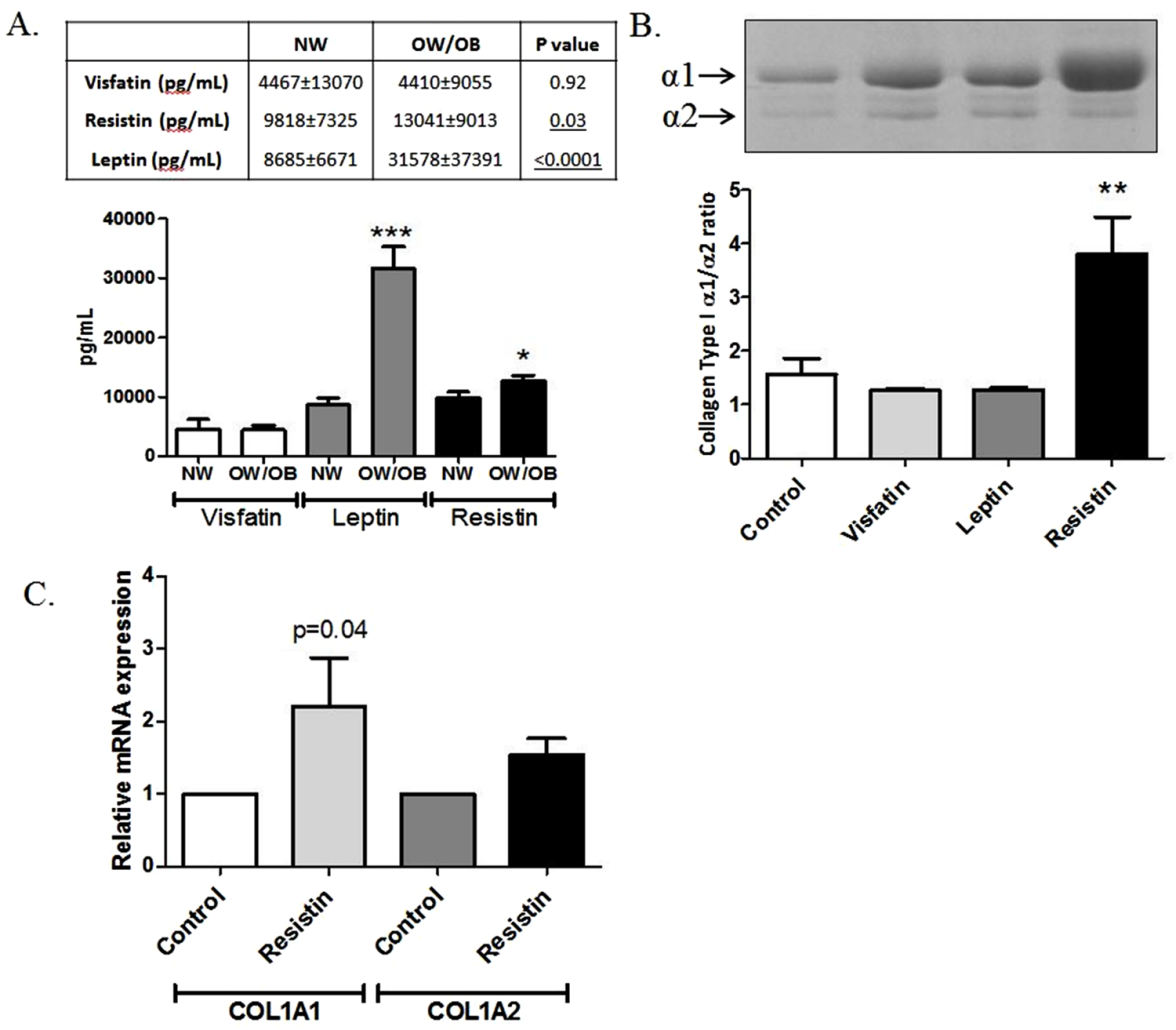
Resistin pathologically alters normal-weight OA bone collagen composition. (**A**) Serum adipokine protein expression in NW (n = 38) and OW/OB (n = 112) patients with OA as determined by Luminex analysis.Values in table represent mean pg/ml ± stdev. Bars in graph represent mean ± SEM. ***p < 0.0001, *p < 0.05. (**B**) The type I collagen *α*/*α* 2 ratio in femoral head subchondral bone explants from NW OA patients following stimulation for 4 weeks with either resistin (500 ng/ml), leptin (100 ng/ml), visfatin (500 ng/ml) or media control. Bars represent mean ± SEM (n = 4). The ratio of *α*1/*α*2 isoforms was quantified by SDS PAGE analysis. Gel image is a representative coomassie-stained gel of subchondral bone explants after 4 weeks of adipokine stimulation. (**B**) The mRNA expression of COL1A1 and COL1A2 following acute (24 h) stimulation of primary hip OA osteoblasts with either resistin (500 ng/ml) or media control. Expression was determined by Sybr Green qRT-PCR, normalised to ACTB. Bars represent mean ± SEM (n = 4).

Following identification of differential collagen formation in the subchondral bone from OW/OB hip OA patients, we next examined whether adipokines could provide a systemic link to altering bone pathology. To this end, we profiled the circulatory concentrations of 21 known adipokines and inflammatory cytokines (namely IL-10, IL-1*β*, DKK1, MIP-1 *α*, Chemerin, Eotaxin, gp130, IP10, MCP1, IL-7, MIP-3 *α*, amphiregulin, IL-15, aggrecan, resistin, adiponectin, IL-6, LIF, leptin, visfatin and MIP-1*β*) in serum samples collected pre-operatively from NW (n = 38) and OW/OB (n = 112) patients with OA undergoing joint replacement surgery (Table 2). Of the adipokines profiled, we identified significant increases in the serum concentration of leptin and resistin in the OW/OB patients, compared to the NW patients (Fig. 2A).

**Table 2:** Patient characteristics of normal-weight (NW, n = 38) and over-weight/obese (OW/OB, n = 112) OA patient cohorts.

Parameter	NW	OW/OB	p value
Gender (M:F)	16:22	60:52	—
Age (yr)	67 ± 9	68 ± 8	0.385
BMI	23.2 ± 1.1	31.5 ± 5.1	**0.0001**
W:H ratio	0.87 ± 0.08	0.93 ± 0.08	**0.005**
Body Fat %	24.6 ± 10.1	34.8 ± 9.2	**0.0001**

W:H ratio = waist circumference:hip circumference. Values represent the mean ± SD.

We then examined whether chronic stimulation of NW OA subchondral bone tissue with either leptin, resistin or visfatin could pathologically alter the composition of Type I collagen to resemble an OW/OB bone phenotype. Bone samples were cultured for 4 weeks in either media containing recombinant leptin (100 ng/ml), resistin (500 ng/ml) or visfatin (500 ng/ml) or in culture media alone (control). After 4 weeks, bone tissue was digested and Type I collagen composition determined by quantifying the relative expression of *α*1 and *α*2 using SDS PAGE. Stimulation of NW bone tissue with either leptin or visfatin had no significant effect on the Type I collagen *α*1/*α*2 ratio. However, NW human bone explants stimulated with resistin exhibited a significant 2.4 fold increase in the Type I collagen *α*1/*α*2 ratio, compared to bone cultured in control media (Fig. 2B, p < 0.01). This effect was further confirmed by a 2.2 fold increase in COL1A1 gene expression following a 24 h resistin stimulation, with little increase in COL1A2 gene expression (1.5 fold) (Fig. 2B).

### Resistin induces activation of the canonical Wnt signalling pathway in primary osteoblasts isolated from hip OA patients and induces the metabolic activity and bone nodule formation in primary osteoblasts isolated from hip OA patients

**Figure 3:**
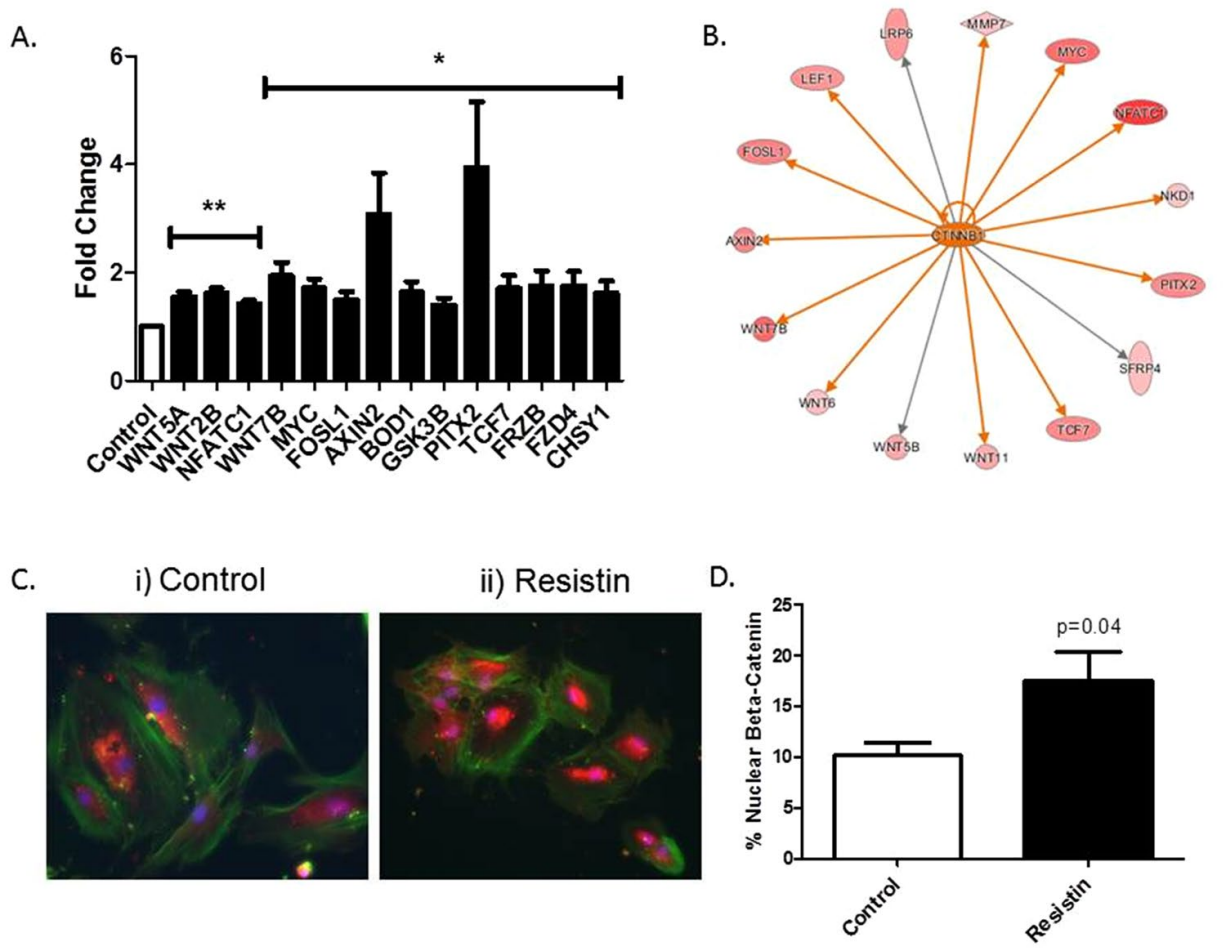
Analysis of the Wnt signalling pathway in human primary osteoblasts following resistin stimulation. (**A**) Canonical Wnt signalling pathway gene expression following resistin stimulation. Primary human osteoblasts cells were stimulated with 500 ng/ml recombinant resistin for 24 hours prior to gene analysis, with 14 genes shown to be up-regulated (**p < 0.01, *p < 0.05). (**B**) IPA analysis of Wnt genes differentially expressed >1.4 fold change with resistin stimulation and the predicted increase in *β*-catenin activity and expression. Orange arrows = predicted activation, grey arrows = effect not predicted. (**C**) Representative images of nuclear translocation of *β*-catenin in human osteoblasts following i) media only ii) 30 min of resistin stimulation (500 ng/ml). Green = *α*-Actin, Blue = Hoechst, and Red = *β*-Catenin (n = 3). (**D**) Quantification of nuclear translocation of *β*-catenin in human primary osteoblasts using Image J software. Percentage nuclear *β*-catenin refers to the subset within the area of hoechst staining compared to the whole cell and is an average of 6 images from 3 OA patient cell lines (n = 3).

Mouse models and several genome wide association studies have implicated canonical Wnt signalling in mediating bone homeostasis^24–26^. Therefore, we next examined the effect of resistin on the Wnt pathway in primary human hip OA osteoblasts cultured from femoral head bone chips. Firstly, we determined the effect of 24 h resistin stimulation on the expression of 84 genes related to Wnt-mediated signal transduction by qRTPCR. Of the 84 genes analysed, 14 genes were found to be significantly modulated by >1.4-fold by stimulation with resistin (Fig. 3A). Next, we performed pathway analysis of these differentially expressed genes using Ingenuity Pathway Analysis software in order to determine the predicted activation status of the Wnt pathway signal transducer, *β*-catenin. Core functional analysis predicted an increase in activity and expression of *β*-catenin (Fig. 3B). To confirm this, we then conducted histochemical analyses of *β*-catenin localisation in primary osteoblasts. Stimulation of osteoblasts for 30 min with recombinant resistin induced nuclear translocation of *β*-catenin, as demonstrated by an increase in nuclear staining of *β*-catenin in resistin stimulated osteoblasts, compared to non-stimulated media control osteoblasts (Fig. 3C and D).

It is widely accepted that *β*-catenin activation is associated with osteoblastic differentiation^27^. Therefore, we next examined whether resistin affected the metabolic activity of primary human OA osteoblasts and their innate ability to form mineralised bone nodules.

**Figure 4:**
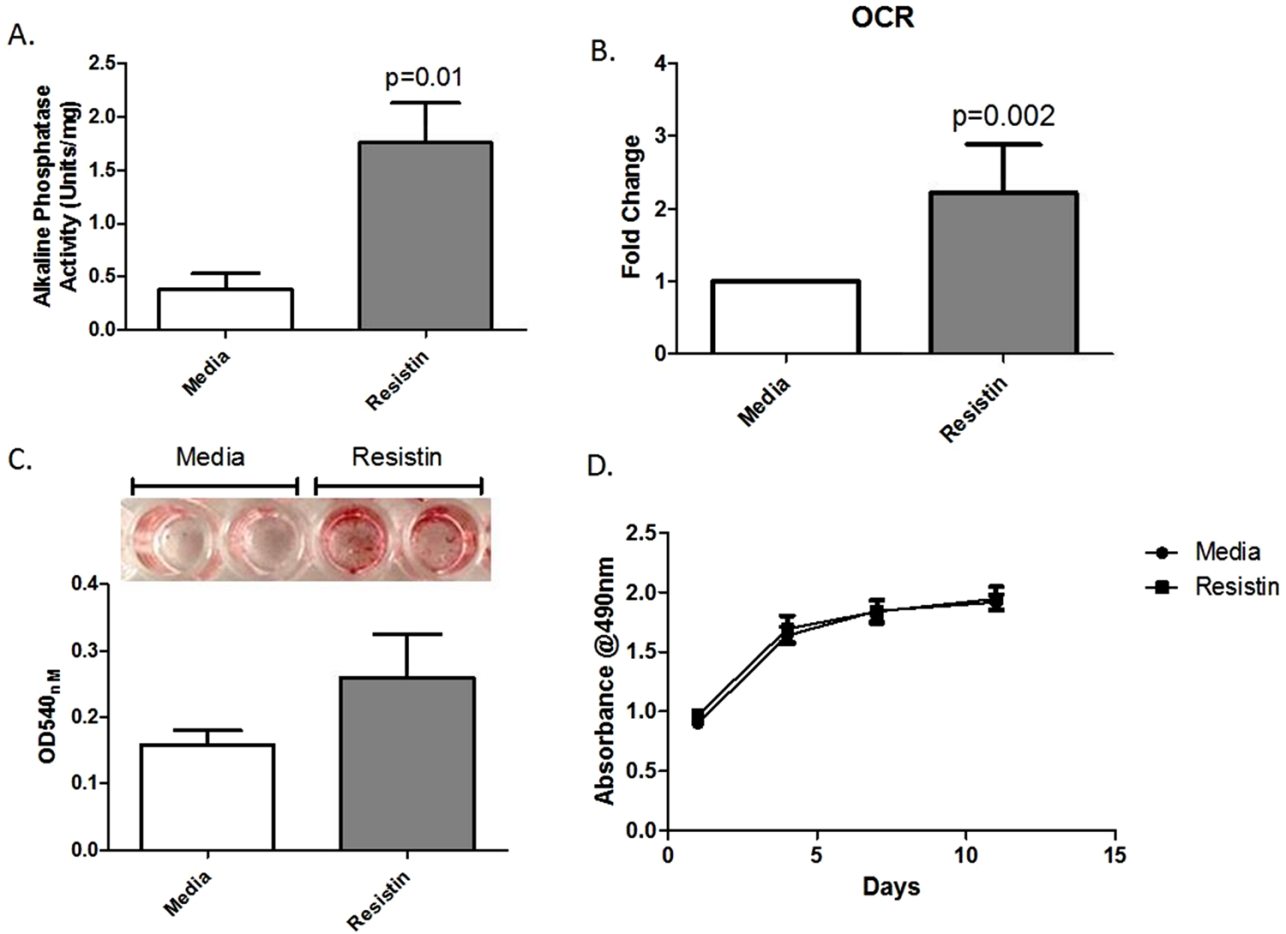
The functional impact of resistin on the metabolic activity and bone nodule formation of human primary OA osteoblasts. (**A**) Alkaline phosphatase activity of human primary OA osteoblasts from the femoral subchondral bone following 2wk stimulation with either resistin (500 ng/ml) or media control. Bars represent mean values ± SEM (n = 4). (**B**) The fold change in the oxygen consumption rate (OCR) following 24 h stimulation of primary human OA osteoblasts with resistin (500 ng/ml), compared to non-stimulated osteoblasts. OCR values (pmol/min) were normalized to total protein following cell lysis prior to fold change calculation. Bars represent mean values ± SEM (n = 10). (**C**) Representative image of alizarin red stained mineralised bone nodules following 4 wk stimulation of primary hip OA osteoblasts with either resistin (500 ng/ml) or media control. Alizarin red staining was quantified using Image J software. Bars represent mean values ± SEM (n = 4). (**D**) Time-course of human primary hip OA osteoblast cell proliferation following stimulation with and without resistin (n = 4).

Primary human hip OA osteoblasts were cultured in either media containing resistin (500 ng/ml), or media alone for up to 28 days. After 14 days of culture, alkaline phosphatase activity was measured and was found to be significantly higher in osteoblasts stimulated with resistin, compared to control osteoblasts cultured with media alone (1.78 ± 0.37 vs. 0.37 ± 0.12 Units/gram; Fig. 4A), suggesting an increase in osteoblast metabolic activity. This was confirmed by our finding that stimulation of osteoblasts with resistin increased their basal oxygen consumption rate (OCR) (Fig. 4B).

Finally, we examined whether this resistin-induced greater metabolic phenotype affected the ability of osteoblasts to form bone by using Alizarin Red to stain and quantify mineralised nodules. Osteoblasts stimulated with resistin for 4 weeks showed greater bone nodule formation (Fig. 4C), compared to osteoblasts cultured with media alone. Of note, we found no effect of resistin on the proliferation of osteoblasts (Fig. 4D).

## Discussion

This is the first study to examine the relationship between adiposity and subchondral bone structural composition in patients with hip OA, and to demonstrate that resistin promotes an abnormal Type I collagen phenotype and alters the metabolic and functional activity of primary hip OA osteoblasts.

Collagen structure and alignment is pivotal to the structural integrity of bone^22, 28^. Therefore, increased proportion of type I collagen homotrimer in OA bone has negative mechanical consequences^22^. In 2002, Bailey *et al*. demonstrated an increase in the relative proportion of *α*1 to *α*2 Type I collagen isoforms in OA subchondral bone when compared to fracture neck of femur patients, resulting in poorly organised collagen matrix, reduced mineralisation of the bone, and increased lysine hydroxylation^22^. The *α*2 chain is thought to be integral to maintaining the triple helix structure of collagen^29^, increasing the interaction of the hydrophobic side chains and thus limiting water content, and allowing for greater cross-linking of the collagen molecules^30^.

In this paper, we found that the *α*1/*α*2 type I collagen ratio was greater in overweight/obese patients with hip OA, compared to hip OA patients of normal-weight. This suggests that adiposity may be a primary driver of collagen malformation, leading to accentuated sclerotic bone development and OA progression^22^.

Structural abnormalities of bone in relation to adiposity were further confirmed via micro-CT. In particular, we have demonstrated a significant increase in BS/BV in the subchondral bone of over-weight and obese hip OA patients; an indication of higher rates of bone turnover occurring as a result of greater bone surface area^31^. In addition, in this study we have shown a decrease in the trabecular thickness of the subchondral bone in hip OA patients who are over-weight/obese, compared to normal-weight patients. This result was surprising since an increase in trabecular thickness has previously been reported in OA subchondral bone, compared to non-OA bone^32^. However, previous studies on subchondral bone trabecular thickness have not compared OA patients of different adiposity and have been conducted in patients with knee OA^33^, which is less susceptible to mechanical stresses than the hip joint^34^. Importantly, dietary-induced obesity in the mouse has been shown to lead to a reduction in femoral head trabecular thickness^35^. Furthermore, Buckland-Wright^36^ suggested that in advanced OA, the greater deformity of articular surfaces leads to greater absorption of local stresses and thus reduced load transmission into deeper sub-articular regions resulting in localised osteoporosis^36^. The differences in trabecular thickness we have observed within our study could be due alterations in hip alignment and force transduction, or in reference to Buckland-Wright^36^, could be due to greater deformity of the articular cartilage due to the excessive load-bearing evident in over-weight/obese individuals^36^.

Given that bone is a highly vascularized tissue, and thus susceptible to pathological levels of circulatory adipokines, our findings on the functional effects of resistin in this study are significant. Resistin, is a dimeric protein, which is associated with insulin resistance in mice^37, 38^. It is secreted from both adipocytes and macrophages and is abundantly expressed in bone marrow cells^39^. In rheumatoid arthritis, resistin has been shown to accumulate within inflamed joints, and to correlate with the degree of inflammation and the expression of inflammatory cytokines including TNF-*α*, IL-1*β* and IL-6. Furthermore, resistin has previously been reported to promote the proliferation of the murine osteoblast precursor cell line, MC3T3-E1^19^. However, despite these findings, very little is understood with regards to the role of resistin in bone pathology, its effect on primary OA osteoblasts and its overall impact on bone remodelling and collagen formation in OA.

In the present study, we found that circulatory levels of both resistin and leptin were significantly elevated in over-weight/obese OA patient serum compared to normal-weight patients with OA. The association of leptin and resistin with obesity is in support of previous research^16, 40–42^. However, we have provided evidence that resistin, unlike leptin, significantly affects the type I collagen composition of subchondral bone, driving the phenotype of normal-weight bone towards a sclerotic homotrimer-rich type I collagen phenotype, consistent with that exhibited by over-weight/obese bone.

Stimulation of primary OA osteoblasts with resistin increased the activation of the canonical Wnt signalling pathway, as indicated by the rapid nuclear translocation of *β*-catenin and the induction of multiple Wnt-mediated signal transduction genes. Critically, the Wnt pathway is believed to be a principal pathway in bone remodelling. It is a known regulator of both alkaline phosphatase activity and osteoblastic differentiation^43, 44^. Furthermore, mutations in Wnt signalling mediators such as the Wnt receptor LRP5 have been associated with abnormal bone mass^45, 46^. Therefore, the current finding that resistin activates the Wnt pathway in primary OA osteoblasts further supports the role of resistin in driving pathological bone formation in obese patients with hip OA.

Further analysis on the role of resistin in bone demonstrated that resistin directly affects the activity and function of primary human OA osteoblasts. Similarly to the effect previously reported of resistin on murine osteoblast precursors^19^, we found that resistin increased alkaline phosphatase activity following a 14 day stimulation of osteoblasts. Importantly, dysregulated alkaline phosphatase activity has also been associated with bone pathologies^47, 48^, and greater alkaline phosphatase activity has been reported in osteoblasts from OA bone. Mature osteoblasts exhibit higher alkaline phosphatase activity. Therefore, it has been speculated that the elevated alkaline phosphatase activity in OA osteoblasts could reflect increased differentiation of pre-osteoblasts into mature osteoblasts^49, 50^. This is further supported by the increased metabolic rate we have demonstrated.

Consistent with the resistin-mediated induction of osteoblast metabolic activity, we also observed that chronic stimulation with resistin increased osteoblast bone nodule formation. This finding appears counterintuitive to previous studies that have shown a negative correlation with serum resistin levels and bone mineral density^51, 52^. However, resistin has been shown to increase cytosolic calcium in human hepatic stellate cells^53^. Importantly, in osteoblasts it has been shown that increases in cytosolic calcium can contribute to increased extracellular hydroxyapatite formation^54^. Therefore, our finding that resistin drives bone nodule formation could be due to increases in cytosolic calcium in osteoblasts.

Of note, we observed no effect of resistin on the proliferation of human primary OA osteoblasts. Therefore, the positive induction of bone nodule formation in osteoblasts stimulated with resistin appears more likely to reflect an effect of resistin on osteoblast maturation and osteoid development. In this context, it was recently reported that resistin, as well as the adipokines visfatin and adiponectin are present in knee osteophytes^55^. Of interest, Junker *et al*. found that adiponectin was found to induce p38 MAPK signalling in primary knee OA osteoblasts, suggesting that adipokines may also influence osteophyte development by modulating proinflammatory conditions^55^.

It is important to note that all the joint tissue samples used in this study were obtained peri-operatively from hip OA patients undergoing total joint replacement suffering from late-stage disease, where typically there is substantial articular cartilage loss. It is inherently difficult to obtain bone tissue from patients with early stage hip OA disease. Therefore, our dataset excludes any patients with less severe OA where the femoral head subchondral bone would be expected to have greater cartilage coverage.

To conclude, our data has demonstrated that adiposity affects the structural composition of the femoral head subchondral bone in patients with hip OA, indicative of an increase in Type I collagen homotrimer content. Furthermore, we have shown that the adipokine resistin is elevated in over-weight/obese OA patients, compared to normal-weight OA patients and promotes an obese subchondral bone phenotype. The effect of resistin on the phenotype of primary human OA osteoblasts, and its activation of canonical Wnt signalling, stresses the importance of continuing to investigate the role of adipokines in OA pathology, particularly in relation to those patients with OA who are over-weight or obese.

## Methods

### Ethical Approval and Subject Recruitment

All experiments and methods were performed in accordance with relevant guidelines and regulations. All experimental protocols were approved by a named institutional/licensing committee. Specifically, the University of Birmingham, UK Research Ethics committee granted ethical approval (NRES 14-ES-1044), in accordance with the principles of Good Clinical Practice guidelines. Participants were recruited on a volunteer basis, after being fully-informed of the study requirements by the clinical research staff of collaborating hospitals. Consent was obtained from all patients for study participation and any identifying information removed from images in this publication. Femoral heads were collected at the time of surgery from hip OA patients undergoing total hip arthroplasty at The Royal Orthopaedic Hospital, Birmingham (UK) or Russell’s Hall Hospital, Dudley (UK). Height and weight measurements were recorded to determine BMI, and blood samples were collected pre-operatively from 112 over-weight/obese and 38 normal-weight patients with OA. All study participants were aged between 45 and 80 years of age. All participants demonstrating secondary causes of OA, such as avascular necrosis, Perthes disease, developmental dysplasia, previous acetabular or femoral neck fractures and slipped upper femoral epiphysis, were excluded from the study.

### Serum adipokine profiling by Luminex

To determine the circulatory concentration of adipokines, multiplex technology (Luminex Screening Assay, R and D Systems) was performed according to the manufacturer’s instructions. In brief, OA patient serum samples were diluted to 1:2 in assay buffer, and the concentration of 21 adipokines were quantified using a Luminex 200 instrument (Luminex Corporation, Austin, Texas, USA).

### Molecular composition of bone type I collagen

Quantification of Type I Collagen *α*1/*α*2 composition was performed as previously described by Bailey *et al*.^22^. Briefly, 150 mg of powdered bone was washed in PBS (Sigma, UK) then resuspended in decalcifying buffer (10% (w/v) EDTA, 30 mM TRIZMA base and 4 M guanidine hydrochloride at pH 7.5) and placed on a rotator at 4 °C. Decalcified bone samples were centrifuged at 5000 rpm for 10 minutes, with the insoluble fraction resuspended in pepsin solution (0.5 M acetic acid and 0.5% w/w pepsin (based upon original bone weight), P6887, Sigma) and rotated at 4 °C for 48 hr. Following pepsin incubation, the centrifuged pellet was discarded and pooled supernatants were freeze dried and analysed by gel electrophoresis.

### Micro-CT scanning

Bone cubes (1 × 1 × 1 cm) were cut by the Royal Orthopaedic Hospital pathology service, from the most medial aspect of the femoral head, as shown in Fig. 1B. Bone cubes were washed in acetone and allowed to air dry prior to micro-CT scanning. Gross structural parameters were determined using a Bruker Sky scan 1172 (Bruker Skyscan 1172, e2v technologies plc, Chelmsford, UK). Sample resolution size was set to 9.87 *μ*m, with an exposure time of 200 ms and a rotation step of 0.4°, and no filter was applied. Scans were performed at 49 kV. Approximately 800 scan slices were collected per sample, with 60% beam hardening correction and a ring artefact correction of 5. Reconstruction was performed using the NRecon software version 1.6.2 (SkyScan, e2v technologies plc, Chelmsford, UK).

### Reconstruction and analysis

Post alignment of each image was minor at 0 ± 1. The reconstruction settings were maintained for all samples (Smoothing = 2, smoothing kernel = 0, reconstruction duration per slice 0.18 s). One hundred slices immediately below the cortical bone layer (As shown in Fig. 1B) were isolated and assayed for quantitative analysis. Regions of interest were drawn within the bone area for each sample. Adaptive thresholding was performed (Settings; round kernel, radius 4, constant 0, background dark, pre-threshold on, lower grey threshold 69, upper grey threshold 255), and white speckles were removed (<20 voxels). Finally a despeckle sweep of the 3D space was used to correct for image irregularities and images were analysed using CTVol software (SkyScan, e2v technologies plc, Chelmsford, UK).

### Primary osteoblast and ***ex-vivo*** bone explant culture

Subchondral bone chips were cut from the femoral head using a Friedman Rongeur, and washed three times in DMEM containing 100 U/mL penicillin streptomycin to remove excess fat, blood, marrow, and connective tissue. To isolate primary human osteoblasts, small bone chips (<3 mm^3^) were placed into a 25 cm^2^ vented flask with osteoblast differentiation media (DMEM, 10% FBS, 100 Units/mL Penicillin Streptomycin, 2 mM L-Glutamine, 1% NEAA, 2 mM *β*-glycerophosphate disodium salt hydrate, 50 ug/mL L-Ascorbic Acid, 10 nM Dexamethasone). Bone chips were cultured in a humidified atmosphere of 37 °C and 5% CO2 and left for 5 days before the initial media change. Following 5 days, differentiation media was changed every 3 days, and bone chips were removed once primary osteoblast cell coverage reached approximately 30% confluency. For osteoblast and *ex-vivo* bone explant stimulations, recombinant proteins (Resistin, 500 ng/ml; Leptin 100 ng/ml; Visfatin, 500 ng/ml; all from Cambridge Biosciences) were diluted in primary osteoblast differentiation media. The selected adipokine concentrations were chosen based on previously published studies on adipokine stimulation of cells from the joint. The concentrations used are higher than the systemic adipokine concentrations in order to reflect that the absolute quantity of adipokines exposed to osteoblasts *in vivo* will be greater than in osteoblast *in vitro* studies, due to blood flow through bone tissue^56–60^.

### Gene expression analysis

To determine the effect of resistin on the Wnt signalling pathway, total RNA was isolated from human hip OA primary osteoblasts stimulated with or without resistin (500 ng/ml) for 24 h. The mRNA expression of 84 genes related to Wnt-mediated signal transduction was determined by qRT-PCR using an RT2 Profiler PCR array kit (Qiagen) as per the manufacturer’s instructions. The mRNA expression of COL1A1 and COL1A2 was determined by Sybr Green qRT-PCR using primers designed by Dharmacon (GE LifeSciences, UK).

### Pathway analysis

Pathway analysis was performed using the pathway analysis software application Ingenuity Pathway Analysis (IPA; www.ingenuity.com). Using a cut-off filter of ± >1.4 fold change and significance P < 0.05, genes that were differentially expressed upon resistin stimulation of primary osteoblasts were entered into the IPA software to generate a network map. A core functional analysis was then performed in order to determine the predicted activation status of the Wnt signal transducer beta-catenin.

### Osteoblast bone mineralisation

Human OA osteoblasts were seeded at 6 × 10^3^ cells per well in a 24 well plate and treated with or without adipokine stimulation as described previously. After 14 days, cells were stained with alizarin red solution (0.5% Alizarin Red (Sigma, UK) in 1% ammonia hydroxide at pH 4.5) for 10 min at room temperature and washed with PBS to remove excess stain. Cells were then incubated in 10% cetyl pyridinium chloride (Sigma, UK) for 10 min at room temperature. The supernatant was collected from each well and diluted 1:10 with the 10% cetyl pyridinium chloride and read at OD550 nm on a microplate Reader (Biotek, Elx808).

### Osteoblast alkaline phosphatase activity

Alkaline Phosphatase (ALP) (Human placenta, P3895, Sigma) was diluted to 100 Units/mL in 1 mM MgCl_2_ (P2670, Sigma) and stored at −20 °C. Human osteoblast cells were seeded at 6 × 10^3^ cells per well in a 24 well plate and treated with or without adipokine stimulation as described previously. Osteoblasts were lysed in RIPA diluent buffer ((0.2x RIPA buffer, 1 mM MgCl2) and diluted 1:5 with 1 mM MgCl_2_. Diluted osteoblast lysates were combined with ALP substrate (solution containing pNPP, P7998, Sigma) and incubated at 37 °C for 15 min. The reaction was stopped with the addition of 0.1 N NaOH and read immediately at 405 nm (Biotek, ELx808).

### Osteoblast oxygen consumption analysis

Osteoblasts were seeded at a density of 6 × 10^3^ cells in XFe24-well cell culture microplates (Seahorse Bioscience, North Billerica, MA). Prior to the assay, cells were placed in 600 *μ*l of Seahorse XF Base Medium (pH 7.4, 10% FBS, 4.6 g/L glucose, 2 mM *β*-glycerophosphate disodium salt hydrate, 10 mM Dexamethasone) pre-warmed to 37 °C. The plate was then transferred to a non-CO2 incubator for 1 h. Following calibration, oxygen consumption measurements were performed for basal respiration. Upon completion of the assay, cells were collected in lysis buffer (RIPA buffer; 0.4% protease inhibitor cocktail) and centrifuged for 10 min at 8,000 g and the supernatant was removed for protein determination. Protein concentration was determined using the BCA protein assay (Pierce, UK).

### Osteoblast cell proliferation assay

Proliferation was determined using the CellTiter 96® Aqueous One Solution Cell Proliferation Assay kit (Promega, USA), as per the manufacturer’s instructions.

### Immunocytochemistry of *β*-Catenin localisation in primary hip OA osteoblasts

Osteoblasts were plated in a 24 well plate for 24 h prior to resistin stimulation (as previously stated). Following stimulation, ice cold 4% paraformaldehyde was added to each well for 20 min. Cells were then washed with PBS before incubation for 1 h in vehicle (10% goat serum in PBS and 0.1% Triton X-100). Cells were then incubated in vehicle containing primary antibody *α*-*β*-catenin (AB6302, Abcam, UK) *α*-Actin (AC-40, Sigma, USA) overnight. Following primary antibody incubation, cells were washed and incubated in vehicle and secondary antibody (*α*-Rabbit H+L, Alexa Fluor 555, Pierce, UK) for 1 h with the addition of DAPI (4083, CST, USA). Cells were washed before mounting with Prolong® Diamond Antifade Moutant (Thermofisher).

### Statistical Analysis

Descriptive statistics were tabulated to detail patient characteristics (Mean ± SD). Gaussian distribution was confirmed using Kolmogorov-Smirnov test. A one-way ANOVA with Tukey Post-Hoc was used to determine collagen homotrimer significance. Unpaired t-tests were performed to determine differences in serum adipokine concentrations in patients of different BMI categories and to determine the effect of recombinant adipokines on osteoblasts in culture, using a confidence of >95% to demonstrate statistical significance. To analyse the WNT pathway gene expression all samples were log transformed prior to statistical analysis. All statistical calculations were performed using Graphpad Prism software.
